# Structure and factorial invariance of the Grit-Original scale and convergent validity of the network with job satisfaction and happiness

**DOI:** 10.3389/fpsyg.2023.1234594

**Published:** 2023-08-10

**Authors:** Cristian Ramos-Vera, Juan José Soza-Herrera, Gleni Quispe-Callo, Antonio Serpa-Barrientos, Yaquelin E. Calizaya-Milla, Jacksaint Saintila

**Affiliations:** ^1^Área de Investigación, Universidad Cesar Vallejo, Lima, Peru; ^2^Departamento de Psicología, Universidad San Martin de Porres, Lima, Peru; ^3^Escuela de Psicología, Universidad Nacional de San Agustín de Arequipa, Arequipa, Peru; ^4^Departamento de Psicología, Universidad Nacional Mayor de San Marcos, Lima, Peru; ^5^Facultad de Ciencias de la Salud, Universidad Peruana Unión, Lima, Peru; ^6^Escuela de Medicina Humana, Universidad Señor de Sipán, Chiclayo, Peru

**Keywords:** psychometrics, perseverance, factor invariance, confirmatory factor analysis, factor analysis

## Abstract

**Background:**

The concept of Grit refers to a person’s ability to maintain perseverance and passion in the pursuit of long-term objectives. However, research on the applicability of the Grit-Original scale (Grit-O) in the Latin American context is limited.

**Objective:**

This instrumental design study aimed to analyze the structure of this scale and its factorial invariance in relation to gender, as well as to examine its convergent validity with job satisfaction and happiness.

**Methods:**

A sample of 364 Peruvian workers that were selected through non-probabilistic convenience sampling in 2021.

**Results:**

The results of the confirmatory factor analysis showed that the two-dimensional structure of 12 items presented adequate goodness-of-fit indices. Additionally, the instrument is invariant between men and women. Likewise, the convergent relationship between the Grit scale, job satisfaction, and happiness variables was confirmed, which supports the validity of the instrument in the study context.

**Conclusion:**

The findings of the study confirm that the GRIT-O is a measure with adequate psychometric properties in the Peruvian context.

## Introduction

In recent years, there has been a growing interest in both the scientific community and the business community in investigating the variables that impact the achievement of personal, academic and work-related goals (Collantes-Tique et al., 2021). In the past, it was widely believed that intelligence was the primary and determining factor in achieving success, with the idea that people with higher intelligence quotient would obtain better results (Uribe-Moreno et al., 2022). However, Duckworth et al. (2007) introduced a non-cognitive approach called Grit, which proved to be a promising indicator for predicting performance.

### Grit conceptual definition

Grit is a quality characterized by perseverance and passion in the pursuit of long-term goals (Duckworth et al., 2007; Chaustre-Jota, 2019). In addition, this concept can also encompass the level of determination that a person has to achieve their short-term goals (Eskreis-Winkler et al., 2014). Grit is composed of two main dimensions: consistency of interest and perseverance of effort (Daura et al., 2020). Consistency of interest is related to commitment and passion toward established goals, while perseverance of effort implies the ability to maintain determination in the pursuit of goals over time, even in difficult or challenging situations (Duckworth et al., 2007). In other words, a person with a high level of Grit will show consistent dedication toward their long-term goals, unaffected by distractions or difficulties that may arise along the way. She will persevere in her efforts and remain committed to her goals despite any temptations or challenges that may arise, allowing her to maintain a consistent focus in the pursuit of her aspirations (Verner-Filion et al., 2020).

### Psychometric literature on Grit-Original scale (Grit-O)

Numerous studies have evaluated the reliability and validity of the Grit scale of Duckworth et al. (2007) using various academic databases, such as EBSCO, Scopus, Proquest, Dialnet, Scielo, and Google Scholar. Research on the applicability of the Grit scale in the Latin American context and in the Spanish-speaking population is limited, since it was developed and validated in the United States by Duckworth et al. (2007). This scale, known as Grit-Original (Grit-O), consists of 12 items and two theoretical dimensions, as mentioned above (Daura et al., 2020). In the original validation study, the two-factor structure was confirmed using a sample of 2,235 individuals from eight U.S. universities (Duckworth et al., 2007).

Similarly, in another study conducted by Sigurdson and Petersen (2018), the psychometric properties of the GRIT-O scale were examined in a sample of 597 computer science students from the United States. The results of Sigurdson and Petersen's (2018) study showed that the GRIT-O is a reliable and valid instrument for measuring the two-factor psychometric-theoretic model. In another psychometric study conducted by Van Zyl et al. (2020), the GRIT-O scale was validated in a sample of 311 adults living in the Netherlands. After contrasting 10 factor models, it was found that the bifactor model, which includes two specific factors and a general factor, obtained better fit indices. These findings support the two-factor structure of the GRIT-O scale and also suggest the presence of an underlying general factor related to the concept of stamina or determination in general. In another investigation carried out by Stone and Schmidt (2022), the bifactorial factor model of the GRIT-O scale was examined in a sample composed of 1915 U.S. residents, ranging in age from 14 to 78 years. The results showed that this bifactorial factorial model was more parsimonious and a better fit to the data. In addition, the equivalence of scale measurement according to age and educational level was reported for both those with school education and those with higher education. Similarly, in a study by Li et al. (2023), the psychometric properties of the scale were examined in a general (*n* = 2,140) and university (*n* = 1,935) population sample from the United States, as well as in workers from China (*n* = 675). In this study, four factorial models were compared and it was determined that the bifactor model, which includes two specific factors and a general factor, presented a better fit. In addition, the partial metric model was reported to be invariant according to gender in each of the respective groups.

It is important to mention that, despite the positive findings on the psychometric properties of the GRIT-O scale in some studies, there are also investigations in which its psychometric results have been questioned and modifications have been made in response to low fit indices in the scale structure. For example, a study conducted by Sordia and Martskvishvili (2020) evaluated the adapted version of the GRIT-O scale in a Georgian population using Confirmatory Factor Analysis (CFA). The sample consisted of 431 participants ranging in age from 17 to 66 years. In this study, two second-order factor models were evaluated to analyze the scale structure. The results supported the two-dimensional structure of the GRIT-O scale. In addition, error covariance indices were identified between items 1 and 12, as well as between items 8 and 12. More recently, another cross-cultural study conducted by Van Zyl et al. (2022), considered a sample of 471 American, 361 Hong Kong, and 1,056 European university students, where a better parsimonious structure was determined in the 11-item bifactorial model (two specific factors and one general factor), item 4 was eliminated due to low factorial saturation. In addition, multigroup invariance among the three samples was determined in this investigation with the following indicators RMSEA (Δ < 0.01) and SRMR (Δ < 0.02 for configuration vs. metric; Δ < 0.01).

On the other hand, there are several studies that demonstrate the validity of the GRIT-O in Spanish-speaking participants. In a sample of 303 university students between 20 and 30 years old, the GRIT-O scale was validated to the Spanish language and it was demonstrated through the CFA that the two-factor structure presents adequate factorial fit indices (Barriopedro et al., 2018). In Mexico, a research was conducted whose main objective was to adapt and validate the GRIT-O in 353 adults in the city of Nueva León, the results showed stable adjustment indexes and confirmation of its two-factor internal structure (Marentes-Castillo et al., 2019). Similarly, the AFC of the two-dimensional model of the GRIT-O scale was evaluated in 235 Argentine university students (χ^2^/df = 3.23, GFI = 0.91, AGFI = 0.87, IFI = 0.89, CFI = 0.89, RMR = 0.070, and RMSEA = 0.089), this study reported adequate internal consistency values between dimensions and of the overall scale greater than 0.72 (Tortul et al., 2020).

### Job satisfaction

Grit, according to Daura et al. (2020), is related to multiple convergent psychological constructs that play a fundamental role in goal attainment. These constructs are necessary to sustain perseverance and long-term effort. In addition, it is closely related to job satisfaction, which is defined as the positive attitude of employees toward their work and their work environment in general, and is divided into two domains: intrinsic and extrinsic (Vázquez-Colunga et al., 2021). The intrinsic component of work refers to the experience of the very nature of work, which includes the enjoyment of activities, processes, and the achievement of work goals (Martínez and González, 2022). On the other hand, extrinsic satisfaction refers to stimuli outside the work process, such as salary, benefits, recognition, and promotion opportunities (Boluarte and Merino, 2015). Workers with a high level of Grit tend to exhibit mostly intrinsic job satisfaction, as they are more resilient to adversity in activities and persistence in overcoming obstacles in the workplace (Tandler et al., 2020). Likewise, extrinsic job satisfaction tends to influence perseverance and passion in employees as it guides them to accomplish work goals as a process for obtaining either financial or emotional reward (Mokhber et al., 2018).

According to the study conducted by Dugan et al. (2019), evidence of a correlation between Grit and job satisfaction was found in a sample of 147 workers in the commercial sector in the United States. This suggests that there is a positive relationship between perseverance and long-term effort, characteristics of Grit, and the degree of satisfaction experienced in the work environment. Another study with similar findings included 436 nurses in rural USA, where Grit and job satisfaction presented significant correlation and at the same time predicted job growth (Sellers, 2019), where satisfaction fulfilled the mediating role in the relationship of Grit with job growth. Park and Cho (2019), also reported the relationship between these two variables in a sample of 239 Korean nurses. Likewise, other more recent research reported the significant effect of Grit on job satisfaction in 209 Indian professionals (Agrawal et al., 2022).

### Happiness at work

Another converging variable is happiness at work, which refers to the sense of well-being, satisfaction, and enjoyment that a worker experiences in relation to their job and work environment in general (Fondon et al., 2019; Misra and Srivastava, 2023). Porras Velásquez and Parra D’aleman (2019) mention that happiness at work is the feeling of fulfillment, satisfaction, and meaning that comes from doing work that is enjoyed and that is considered valuable and meaningful. Job satisfaction, according to studies such as those by Fredrickson (2001) and Wagner et al. (2020), is associated with a general disposition to experience positive emotions and to have an optimistic view of both the world and oneself. These aspects strengthen a persevering attitude toward work tasks. In addition, other studies (Goh et al., 2022; Shanmugam and Hidayat, 2022) suggest that workers with a higher level of Grit tend to be more motivated toward the tasks assigned to them, as they feel identified and perceive progress toward the established goals. This has been mentioned within the framework of Seligman’s PERMA model, which addresses the domain of achievement and involves moving toward goals with constant effort and active work (Donaldson et al., 2021; Jimenez et al., 2022). According to these studies, workers who experience greater well-being and happiness tend to experience a greater sense of accomplishment, as they feel satisfied and motivated with the work they do. In addition, they tend to show high levels of perseverance, which promotes a constant determination to achieve established goals (Garn and Simonton, 2020).

Studies between Grit and work happiness have provided results and implications for situational adaptability and growth within the workplace (Datu et al., 2017). Grit and job happiness were shown to be predictors of other psychological constructs such as hope, meaning in life, and subjective well-being (Muhammad et al., 2020). Similarly, it was shown that people who are more goal-oriented tend to be happy and satisfied with their lives, as they use persistence, consistency, and well-being as a foundation for career success (Mazhar Khan and Khan, 2017). In addition, research on 272 workers in Pakistan reported that persistence and consistency of goal achievement was related to happiness at work and psychological capital, where the latter measure had a significant mediating effect on the relationship of the other two variables (Shafique et al., 2023).

Despite the abundant previous research on the tool used to measure the capacity for perseverance and passion to achieve long-term goals in workers, it is necessary to present empirical evidence that takes into account the specific cultural characteristics of the Peruvian context. In particular, it is necessary to consider that the psychometric antecedents of this instrument have been developed mainly in Anglo-Saxon countries, which present significant differences in terms of socioeconomic level and more favorable working conditions, with greater opportunities for promotion and a more individualistic rather than communitarian approach. These cultural differences may influence how measures of perseverance and passion for achieving long-term goals are perceived and responded to in Latin American contexts such as Peru (Sabastizagal-Vela et al., 2020; Vallejo and Giachi, 2021). It is also important to carry out a gender equivalence analysis in the validation of the instrument, in order to reduce any potential bias and allow for adequate comparisons that take into account the specific dynamics of the Peruvian context, which may have a differential influence on men and women.

Based on the arguments presented, it is valid to state that there is a need to use more rigorous methodological tools to compare and understand the factors that influence the performance of workers in various economic sectors, which in turn can inform and support decision making and the implementation of effective interventions to promote a healthy and productive work environment. Therefore, the main objective of this research is to assess the factorial invariance of the GRIT-O scale and to examine its convergent validity with job satisfaction and happiness through statistical relationship network analysis. This will allow us to gain a better understanding of how the Grit relates to job satisfaction and happiness and provide a solid foundation for future research in this area.

## Materials and methods

### Design

The present study adopts a quantitative approach with an instrumental design to evaluate the psychometric properties of the instrument used. Furthermore, it is considered a cross-sectional design as data is collected at a single time point (Cabrera, 2023).

### Participants

The study was conducted using a non-probabilistic convenience sampling technique to select the participants’ sample. A total of 364 Peruvian workers who were part of micro, small, and medium-sized companies and were employed on a payroll or service provision basis. Of these workers, 188 were women (51.6%) and 176 were men (48.4%). Regarding age distribution, participants were in the following ranges: 18–24 years (12.9%, *n* = 47), 25–31 years (53.6%, *n* = 195), 32–39 years (22.3%, *n* = 81), 40–49 years (8.6%, *n* = 32), and 50–59 years (2.5%, *n* = 9). In terms of educational level, the distribution of the sample was as follows: high school (5.8%, *n* = 21), technical (15.7%, *n* = 57), university (22%, *n* = 80), bachelor (23.1%, *n* = 83), bachelor’s (27.5%, *n* = 100), and master’s (6%, *n* = 22).

### Instruments

#### Original Grit scale (Grit-O)

The GRIT-O Scale developed by Duckworth et al. (2007) was used to assess the level of Grit of the participants. In this study, the Spanish adapted version of Tortul et al. (2020) was used, which consists of 12 items in total. The scale is composed of two main dimensions: perseverance of effort (PE) and consistency of interest (CI). The perseverance in effort dimension includes 6 items (1, 4, 6, 9, 10, and 12), which assess the ability to remain committed and persistent in achieving goals despite challenges and setbacks. The consistency of interest dimension also consists of 6 items (2, 3, 5, 7, 8, and 11), which assess the ability to maintain a constant interest and passion toward goals and activities over time. These items were used to measure the level of Grit in the participants, and were scored on a response scale reflecting the degree of agreement with each statement. The response format is Likert-type with five options: 5 = Very much, 4 = A lot, 3 = More or less, 2 = A little, and 1 = Not at all. In this research, the instrument used showed an overall reliability omega coefficient of 0.75. For the CI dimension, an omega coefficient of 0.77 was obtained, while for the PE dimension it was 0.73. These values indicate that the instrument used has a good internal consistency.

#### Job satisfaction scale

In this study, the job satisfaction scale developed by Cooper et al. (1989) and adapted to the Peruvian version by Boluarte and Merino (2015) was used. The version used consists of 15 items in total and assesses two main dimensions: intrinsic satisfaction and extrinsic satisfaction. Both dimensions were used to measure the level of job satisfaction of the participants in this study. Each item was scored on a response scale reflecting the participant’s degree of agreement or satisfaction with each statement. The response format is Likert-type and is: 6 = Very satisfied, 5 = Satisfied, 4 = Moderately satisfied, 3 = Moderately dissatisfied, 2 = Dissatisfied, and 1 = Very dissatisfied. The overall reliability result obtained for this scale was adequate, with an omega coefficient of 0.81. This value indicates that the scale items are consistently and reliably correlated with each other.

#### Happiness at work scale

In this research, the Happiness at Work Scale developed by Lutterbie and Pryce-Jones (2013) was used, which was adapted to Spanish by Gabini (2017). This scale assesses the level of happiness experienced by individuals in the work context and consists of a set of items (9 items) designed to measure different aspects related to satisfaction and well-being at work. Moreover, it is a one-dimensional measurement. Its response format is Likert-type where 5 = Always, 4 = Almost always, 3 = Sometimes, 2 = Almost never 1 = Never. In relation to the reliability and validity analysis of the scale, a high overall consistency was obtained with a reliability coefficient of 0.91, which supports the reliability of the measure to assess the level of happiness at work.

### Procedure

To validate the content of the test, a review process of the scales and their component items was carried out. The purpose of this process is to ensure that the items are relevant and representative of the construct they are intended to measure. Subsequently, a content validation by judges’ criteria was carried out. In this step, a group of 5 experts, who are specialists in the area of study, was convened. These judges reviewed the scale and items individually, evaluating their relevance, clarity, and representativeness in relation to each construct. Conceptual content was also verified using Aiken’s V coefficient, which accepts values greater than 0.70 (Aiken, 1980). In addition, the corresponding authorization was obtained from the authors of the instrument adapted for the Spanish-speaking population to carry out the present study.

According to information provided by the Ministry of Labor (Ministerio de trabajo (Mintra), 2021), the study population for this research was composed of a total of 16,264,000 Peruvian workers of both genders. In order to select the sample of participants in this research, inclusion criteria were established for individuals to meet. These criteria included being workers of Peruvian nationality, being 18 years of age or older, residing in the country and currently employed in companies and as exclusion criteria, not having a valid employment contract and having less than 2 months in their current employment were considered. These criteria made it possible to define the group of individuals eligible to participate in the study and ensured that the participants were representative of the target population. Non-probability purposive sampling was considered to meet the established criteria. Data collection for this research was conducted in 2021 during the first semester through an online form using Google Forms. This choice was due to the health circumstances existing at the time, with the aim of ensuring the safety and health of the participants by avoiding physical contact and face-to-face meetings. In order to ensure ethical data collection, an informed consent process adapted to the online form platform was implemented. In this informed consent, clear details were provided about the objectives of the study, the confidentiality of the data collected, and the requirements to perform the test. In the data collection process, the ethical guidelines established in the Nuremberg Code and the Helsinki Code (Asociación Médica Mundial, 2008) were taken into account. By considering these ethical guidelines, it was ensured that data collection was conducted in a manner that was ethical and respectful of the rights and welfare of the participants.

### Data analysis

The statistical program Jamovi 1.6.23 was used to explore the psychometric properties of the GRIT-O scale. The results obtained were divided into four stages. The first stage consisted of performing a descriptive analysis of the items to evaluate whether the assumptions of multivariate normality were met. This was carried out by calculating the kurtosis and skewness coefficients of the data (Hair et al., 1995). In the second stage of the research, the evaluation of the factorial validity of the GRIT-O scale was carried out using confirmatory factor analysis (CFA). For this analysis, a Weighted Least Squares Mean and Variance adjusted (WLSMV) estimator was used due to the ordinal consideration of the data (Hu and Bentler, 1999). The fit indices used in the study were the ratio of chi-square to degrees of freedom (χ^2^/df < 4; Hair et al., 2014), root mean square error of approximation (RMSEA ≤ 0.08), root mean square standardized residual root (SRMR ≤ 0.08), comparative fit index (CFI > 0.90), and Turkes-Lewis index (TLI > 0.90) (Hu and Bentler, 1999). In the research, the McDonald Omega coefficient was used to assess the reliability of the GRIT-O scale as a whole, as well as in its specific dimensions (Viladrich et al., 2017). The invariance of the GRIT-O scale according to gender was evaluated using a multigroup CFA. In this type of analysis, the aim is to determine whether the factor structure of the scale is equivalent between different groups, in this case, between men and women. The invariance assessment process begins with an unrestricted measurement model, also known as configural invariance. Once the general model is obtained, we proceed with the restriction of the factor loadings to determine the metric invariance, consecutively, we restrict the intersections of the elements to obtain the scalar invariance, and finally, we restrict the residuals to reach the residual invariance. A model is considered invariant if the difference in the CFI is less than or equal to 0.01, and if the difference in the RMSEA and SRMR indices is less than or equal to 0.015 (Chen, 2007). Convergent validity was assessed by network analysis using the statistical program JASP 0.16.1. In this analysis, the relationships between the variables (nodes) including the Grit factors, job satisfaction, and happiness at work were represented. Partial correlation metrics (connections) were obtained, where relationships between nodes can be positive or negative. This network model considers the Fruchterman-Reingold “FR” algorithm (Fruchterman and Reingold, 1991). This algorithm is used to visualize and distribute the nodes in a two-dimensional space in a way that reflects the relationships between them. Furthermore, in the network analysis performed in this study, a 2,000-sample nonparametric bootstrapping approach was applied (Epskamp and Fried, 2018). This combination (Fruchterman-Reingold algorithm and nonparametric bootstrapping of 2,000 samples) allowed us to obtain a more robust and specific network model for the analysis of the relationships between Grit factors, job satisfaction, and happiness at work.

## Results

### Descriptive statistics

Table 1 shows that items P6 and P12 have the highest mean value of all items (*M* = 4.31, SD = 0.68) and (*M* = 4.29, SD = 0.74), respectively. The results for skewness and kurtosis are within the acceptable range of −1.5 to +1.5 for univariate normality (Muthén and Kaplan, 1985).

**Table 1:** Descriptives of the GRIT-O scale.

Items	λ_i_	*M*	SD	g1	g2
P1	0.47	3.97	0.77	−0.525	0.271
P2	0.51	2.57	0.96	0.355	−0.348
P3	0.50	2.83	0.98	0.330	−0.354
P4	0.40	3.35	1.35	−0.453	−1.082
P5	0.65	2.27	1.01	0.710	0.090
P6	0.74	4.31	0.68	−0.752	0.430
P7	0.66	2.57	1.15	0.581	−0.466
P8	0.57	2.39	1.02	0.717	0.162
P9	0.69	4.10	0.74	−0.606	0.280
P10	0.59	3.96	0.87	−0.679	0.415
P11	0.44	3.07	0.93	0.045	−0.477
P12	0.78	4.29	0.74	−0.881	0.572

λ_i_, factor loading; M, mean; SD, standard deviation; g1, skewness, g2, kurtosis.

### Confirmatory factor analysis

The CFA was used to test the structure of the two-factor GRIT-O scale, such that the model has acceptable fit indices (χ^2^/df = 3.64, CFI = 0.938, TLI = 0.923, RMSEA = 0.075, SRMR = 0.071), and factor loadings above 0.40 (Bandalos and Finney, 2010). When reliability analyses were performed, an overall Cronbach’s alpha of 0.75 (SD = 0.76; CI = 0.73) and an overall McDonald’s Omega of 0.76 (SD = 0.77; CI = 0.75) were obtained, indicating acceptable reliability, according to the recommended parameters (>0.70, Hayes and Coutts, 2020).

### Factorial invariance

As for the factorial invariance results according to gender, the unrestricted configural model was used as a reference to compare with the other restricted models. The values obtained for these models were considered acceptable according to the following fit indices: CFI = 0.954, RMSEA = 0.043, and SRMR = 0.071. Subsequently, metric invariance was found to have adequate fit indices (CFI = 0.945, RMSEA = 0.045, SRMR = 0.075). Likewise, scalar invariance evidenced fit indices (CFI =0.957, RMSEA = 0.034, SRMR = 0.071) and, finally, residual invariance was demonstrated (CFI = 0.954, RMSEA = 0.043, SRMR = 0.071, ΔCFI ≤ 0.003, ΔRMSEA ≤ 0.009 and ΔSRMR ≤ 0.000), which corroborates the invariance between both group categories (see Table 2).

**Table 2:** Factorial invariance of the GRIT-O scale.

Invariance	*x*^2^/df	CFI	RMSEA	SRMR	∆CFI	∆RMSEA	∆SRMR
Configural	1.339	0.954	0.043	0.071	–	–	–
Metric	1.362	0.945	0.045	0.075	0.009	0.002	0.004
Scalar	1.224	0.957	0.034	0.071	0.012	0.009	0.004
Residual	1.223	0.954	0.043	0.071	0.003	0.009	0.000

χ^2^/df, Chi-square between degrees of freedom; CFI, Comparative Fit Index; RMSEA, Root Mean Square Error of Approximation; SRMR, Standardized Residual Mean Ratio.

### Convergent validity

**Figure 1:**
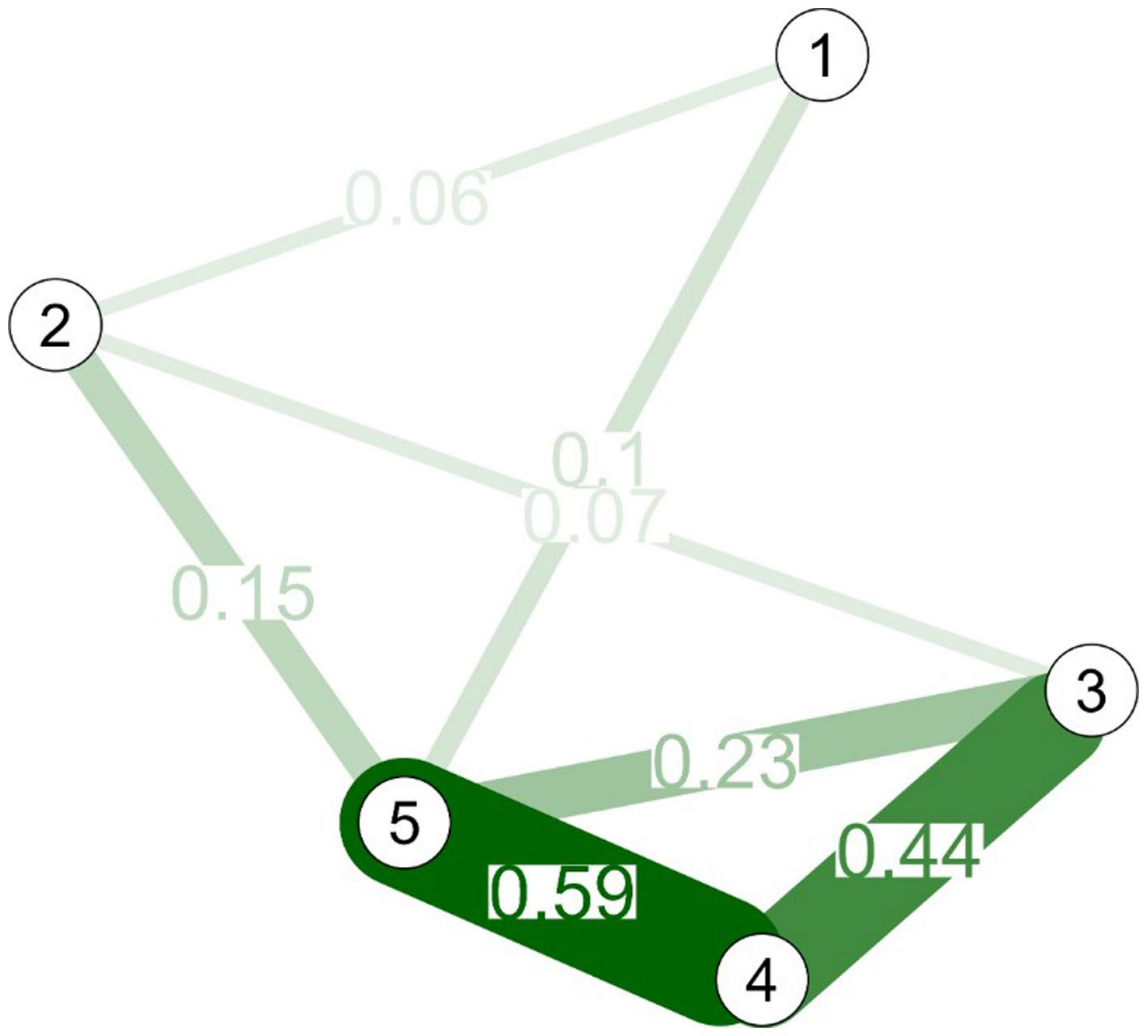
Network analysis of the variables. 1: consistency of interest; 2: perseverance in the effort; 3: intrinsic satisfaction; 4: extrinsic satisfaction; 5: work happiness.

In Figure 1, convergent validity was reported through the analysis of psychometric networks with partial correlations, where it is observed that the perseverance in effort dimension had a direct relationship of greater intensity with happiness (partial *r* = 0.15) and extrinsic satisfaction (partial *r* = 0.07). Likewise, happiness has a direct relationship with consistency of interest (partial *r* = 0.10). The extrinsic satisfaction dimension presented the highest expected influence centrality (IE = 1.07), which refers to the influence of interconnection with the other variables. In addition, perseverance in effort and consistency of interest had an EI of −0.867 and − 1.254, respectively.

## Discussion

There are a variety of personal attributes that can be cultivated and enhanced over time to achieve success in various areas of life, such as education, employment, sports, and creativity. The concept of Grit encompasses the belief that success can be achieved through effort and perseverance, as well as the ability to regulate emotions and self-control, and the ability to set realistic and achievable goals (Li and Li, 2021). Although the GRIT-O scale is widely used to evaluate these aspects, its validation in Peruvian workers in various sectors is still limited. Consequently, the main objective of this study was to adapt the GRIT-O scale in a sample of Peruvian workers and to evaluate its psychometric properties in a work context. The results obtained in this study provided solid evidence of reliability, internal validity, and convergence of the GRIT-O scale with related variables such as happiness and job satisfaction. The GRIT-O scale is used to measure Grit, which is the combination of passion and perseverance toward a long-term goal (Duckworth et al., 2007). This instrument is composed of 12 items and two dimensions: perseverance in effort and consistency of interest, and each is composed of six items (Tortul et al., 2020). These findings support the suitability and usefulness of the scale as a valid and reliable tool to measure perseverance and passion for long-term goals in the Peruvian work context.

The validation of this instrument was based on the internal structure carried out using the CFA, which reported adequate fit indices. These results are in line with previous studies conducted with Spanish-speaking participants, which have investigated the GRIT-O scale in different work contexts. These studies have included samples of workers from Colombia (Collantes-Tique et al., 2021), Mexico (Collantes-Tique et al., 2021), and Spain (Barriopedro et al., 2018; Barrientos Oradini et al., 2022). Consistent findings in different Spanish-speaking countries support the generalizability and robustness of the GRIT-O scale in the workplace, providing a solid basis for its use in research related to the development of perseverance and passion for goals at work. It is important to note that these studies were conducted prior to the COVID-19 quarantine period. Therefore, the present study has the particularity of evaluating the validity of the instrument in a post-pandemic context and associated confinement measures. This is particularly relevant since the pandemic has had a significant impact on various aspects of work and personal life, which may influence workers’ attitudes, behaviors, and perceptions (León-Paucar et al., 2021). Therefore, assessing the validity of the instrument in this post-pandemic context provides up-to-date and relevant information on the applicability of the GRIT-O scale under changing and challenging conditions.

Similarly, the bifactor structure of the GRIT-O scale has been confirmed in different cultural contexts and languages, which supports its cross-cultural validity. An example of this is the study by Li et al. (2023), who validated the scale in Chinese workers. A recent study considered 297 young people in India as participants (Singh and Chukkali, 2021). Furthermore, in another study, this instrument was adapted in health care workers in South Korea (Song and Lim, 2020). Furthermore, in the African context, previous studies have been conducted that have shown satisfactory results in terms of the psychometric parameters of the GRIT-O scale. These studies included professional athletes from Egypt (Shaban, 2020) and university students from Ghana (Stephen Lenz et al., 2018). In the European context, favorable results regarding the fit of the bifactorial structure of the GRIT-O scale have been found in countries such as Georgia and the Netherlands. Previous studies conducted in these countries, such as that of Sordia and Martskvishvili (2020) in Georgia and Van Zyl et al. (2020) in the Netherlands, have supported this bifactor structure of the scale. Through these studies, the versatility and adaptability of the GRIT-O scale in diverse cultural and work environments is evidenced. These studies contribute to the expansion of the knowledge base on the Grit construct and its application in different international contexts, allowing for broader comparisons and analyses of perseverance and success in different populations.

This study indicates that the GRIT-O scale demonstrated adequate reliability using the Omega coefficient. Similarly, a systematic review by Abu Hasan et al. (2020) analyzed the reliability of 20 instrumental investigations from 2015 to 2020 in populations in Europe, North America, Asia, and Africa. It was evident that all studies reported internal consistency through Cronbach’s alpha of the overall scale, which ranged from 0.59 to 0.85 (Datu et al., 2015; Zhong et al., 2018). In eight previous studies that were conducted in 11 countries, the reliability of the GRIT-O scale was assessed and values ranging from 0.75 to 0.92 were found. Most of these studies showed higher reliability compared to the present study, including one paper that was conducted in Spanish-speaking participants (Marentes-Castillo et al., 2019). Overall, most studies reported higher levels of reliability than those found in the present study. It is important to note that the mentioned researches only applied Cronbach’s alpha reliability, while the use of the Omega coefficient presents greater robustness and takes into account the variance of the items, which may result in more accurate estimates of the reliability of the instruments (Viladrich et al., 2017).

Likewise, it was identified that the GRIT-O scale is invariant according to gender, at the configural, metric, scalar, and residual levels. It should be noted that this analysis is highly significant and transcendental in the Latin American context, since no previous studies have been found that report the measurement invariance of the instrument in question, especially at all restrictive levels. Although a study has been conducted in the Spanish-speaking population that examined the invariance according to gender in the two-dimensional structure of the GRIT-O scale, it is important to note that only the first level of invariance, known as configurational invariance, was reported. This study was conducted in a sample of 303 Spanish participants (Barriopedro et al., 2018).

On the other hand, factorial equivalence has also been studied in a bifactor structure, which includes a general dimension and two specific dimensions: perseverance in effort and consistency of interest. In a recent study, Li et al. (2023) examined factorial invariance by gender in two samples: a general U.S. population of 2,140 participants and 675 Chinese employees. The results showed that only configural invariance was confirmed, both in the cross-cultural context between the U.S. samples (general population and university students) and the Chinese sample. In addition, a psychometric study conducted by Van Zyl et al. (2022) demonstrated multigroup (scalar) invariance among university students from different contexts. This study involved 471 students from the USA, 361 from Hong Kong, and 1,056 European participants. However, an 11-item factor structure was used in this study, as item 4 was removed from the scale. Additionally, multigroup invariance of the GRIT-O bifactor model according to age (14–19, 20–29, 30–39, 40–49, and 50–78) and educational level (high school, college degree, and graduate degree) has been reported. This study was conducted on 1,915 US participants by Stone and Schmidt (2022).

In relation to convergent variables, significant relationships were found between perseverance in effort and happiness at work. When workers strive to achieve important work goals despite difficulties, this can generate a sense of purpose and meaning in their work that goes beyond the salary (Garn and Simonton, 2020). This sense of purpose can contribute to an enhanced sense of happiness at work. Workers who feel connected to their work and recognize their importance within the organization are more likely to experience high levels of happiness at work. Garn and Simonton (2020) support this idea by highlighting the positive relationship between striving for work goals and the perception of purpose and meaning at work, which translates into greater happiness in the workplace. Specifically, perseverance in effort helps collaborators to overcome obstacles and challenges at work, which can give them a sense of pleasure and happiness by identifying that their persistence generates significant progress in achieving work objectives (Porras Velásquez and Parra D’aleman, 2019). Item 1 of the instrument (“I have overcome adversity to achieve an important challenge”) shows a close relationship between the concept of perseverance in effort and happiness at work. According to Datu (2021), when workers face and overcome difficulties in the pursuit of meaningful challenges, they experience greater psychological well-being. This capacity for self-improvement and resilience is associated with a greater sense of happiness in the work environment. In other words, workers who face adversity and overcome it in pursuit of important goals tend to experience greater satisfaction and happiness in their workplace.

Likewise, the relationship between perseverance in effort and extrinsic satisfaction was identified, these findings are explained due to the fact that workers feel satisfied with external elements, so they tend to be more perseverant at work, given an opportunity to obtain a reward, whether economic or emotional (Mokhber et al., 2018). For example, if a worker feels satisfied with his salary, he is more likely to feel motivated to fulfill his work objectives, since he knows that his work is adequately rewarded, as referred to in item 10 (“I have achieved a goal or objective that took me years of work”). Additionally, this factor of perseverance in effort is related to extrinsic job satisfaction. This is because when an individual stays focused on his or her aspirations and overcomes obstacles to achieve important goals, he or she experiences a sense of accomplishment. This achievement is perceived externally through social recognition by peers and family members, as well as through monetary rewards or possible job promotions. These external rewards reinforce the worker’s motivation and willingness to carry out other work activities (Boluarte and Merino, 2015).

Likewise, the dimension of consistency of interest also presents a significant relationship with happiness at work. This is because workers who show greater commitment and interest in their work activities experience satisfaction with their performance and the results they obtain in the workplace. This sense of job satisfaction and achievement contributes to greater happiness and positive emotions in the work environment (Shafique et al., 2023). Both constructs have a reciprocal relationship. For example, if a worker presents high levels of consistency of interest in their work activities, it is likely that they tend to perceive and attach a more significant and closer value to their work, which increases feelings of work happiness and well-being; on the other hand, individuals who feel happy in their jobs may be more motivated and committed to achieving long-term goals (Álvarez, 2022).

### Limitations and future research

The present study has a cross-sectional design, which implies that data were collected at a single point in time. This limitation may affect the ability to establish causal relationships and the predictive validity of the instrument. To improve the evidence regarding the predictive validity of the instrument, future longitudinal research is suggested. These studies would involve following participants over time, collecting data at different points in time and assessing how scores on the GRIT-O scale relate to long-term outcomes, such as academic performance, job success, or achievement of personal goals. Furthermore, it is suggested that a test–retest analysis be performed in future research to evaluate the reliability of the instrument. This analysis would involve administering the scale to a group of participants at two different times, with a sufficient time interval between measurements. This would allow us to evaluate the stability of the scores on the scale and to determine whether the instrument produces consistent results over time. In addition, it is important to take into account the representativeness of the sample selected in the present study. Although it is mentioned that the sample was diverse, it is essential to consider the need to obtain a sample that is as representative as possible of the target population, which would allow the results of the study to be generalized to the broader population. In fact, the inclusion of a larger sample in future research may also increase the external validity of the study, providing greater variability and allowing for more robust and generalizable analyses.

## Conclusion

The findings of this study support the validity and reliability of the GRIT-O scale in assessing consistency of interest and perseverance in effort in workers from different economic sectors. The results indicate that the scale is an adequate and valid tool to measure these aspects in this population. In addition, the structure of the scale was found to be gender invariant, suggesting that the instrument maintains its structure and psychometric properties for both men and women in the context of workers in various economic sectors. Moreover, this study also demonstrated the convergent validity of the scale by finding a significant relationship between the GRIT-O scale scores and the variables of job satisfaction and happiness. Future research is suggested to deepen the study of the psychometric properties of the scale in different regions and contexts.

## Data availability statement

The original contributions presented in the study are included in the article/supplementary material, further inquiries can be directed to the corresponding author.

## Ethics statement

The studies involving human participants were reviewed and approved by Research Ethics Committee (Registration Code: N°DR-0023-P-20). The patients/participants provided their written informed consent to participate in this study.

## Author contributions

CR-V was in charge of the project as the principal investigator. JJS-H and GQ-C participated in the study design. YEC-M, AS-B, and CR-V collaborated in the survey design, data collection, and analysis. GQ-C and JS wrote the first draft of the manuscript. All authors contributed to the article and approved the submitted version.

## Conflict of interest

The authors declare that the research was conducted in the absence of any commercial or financial relationships that could be construed as a potential conflict of interest.

## Publisher’s note

All claims expressed in this article are solely those of the authors and do not necessarily represent those of their affiliated organizations, or those of the publisher, the editors and the reviewers. Any product that may be evaluated in this article, or claim that may be made by its manufacturer, is not guaranteed or endorsed by the publisher.
